# Primary Aortoesophageal Fistula Presenting as Upper Gastrointestinal Bleed

**DOI:** 10.7759/cureus.30018

**Published:** 2022-10-07

**Authors:** Naveen Naik, Oseen Shaikh, Muhsina Kunjumohammed, Gopal Balasubramanian

**Affiliations:** 1 Surgery, Jawaharlal Institute of Postgraduate Medical Education and Research, Puducherry, IND

**Keywords:** hematemesis, herald hemorrhages, chiari’s triad, aortoenteric fistula, aortoesophageal fistula

## Abstract

Aortoenteric fistula is a rare condition that can be primary or secondary. The primary type is less common than the secondary. The secondary aortoenteric fistula is an uncommon fatal complication after reconstructive surgery for an aortic aneurysm or other aortic diseases. Here we present a case of a 59-year-old man who presented to our emergency department with sudden onset of massive hematemesis. Imaging studies were done and the patient was diagnosed to have aortoesophageal fistula. Upper gastrointestinal endoscopy showed an intraluminal bulge in the posterior wall of the esophagus with an ulcer and hematoma. The patient was planned for emergency surgical intervention, but the patient had recurrent bouts of hematemesis and, unfortunately, expired. Hence patients presenting with massive hematemesis, aortoenteric fistula can be the underlying cause, and all surgeons should be aware of such conditions and the need for emergent surgical intervention.

## Introduction

Primary Aortoenteric Fistula (AEF) is a rare entity primarily associated with a pre-existing aortic aneurysm. Primary AEF is a communication between the enteric tract and the aorta which develops spontaneously. Secondary AEF is more common than the primary one, occurring as a postoperative complication of aortic reconstructive surgery [1-4]. It is more common in males, and the annual incidence of primary AEF is around 0.007 per million [4]. The clinical features of AEF include hematemesis, generalized abdominal pain, melena, and sepsis, but the condition also may also be clinically occult. Usually, esophagogastroduodenoscopy (EGD) is preferred in a hemodynamically stable patient. In hemodynamically stable patients, contrast-enhanced computed tomography (CT) is the imaging modality of choice because of its high efficiency and widespread availability for evaluating AEF in the acute setting [2]. Computed Tomography Angiography (CTA) and Magnetic Resonance Imaging (MRI) are helpful for surgical planning. Surgery is the mainstay of treatment, which includes in situ reconstructions and extra-anatomic bypass with aortic ligation [3]. Complications of AEF include hemoperitoneum or hemomediastinum and hemorrhagic shock, causing the patient's sudden death in untreated patients. Here we present a case of a 58-year-old male presented with hematemesis, diagnosed to have primary aortoesophageal fistula. The patient succumbed due to massive hematemesis. 

## Case presentation

A 59-year-old male presented to our casualty with complaints of hematemesis of three episodes for one day; each episode contained around 100 mL of fresh blood. He had no similar complaints in the past. He had no prior history of hospitalization for any medical or surgical condition. He had no history of haematochezia, melena, or hemoptysis. He had no previous surgery, trauma, peptic ulcer disease, drug abuse, or other medical conditions. No history of drug intake, diabetes, or hypertension. He was a smoker and occasional alcoholic for 30 years. On examination patient's blood pressure was 124/70 mm of Hg, with tachycardia of 104 beats per minute. The abdomen was soft and non-tender. A review of other systems was otherwise non-significant.

**Figure 1:**
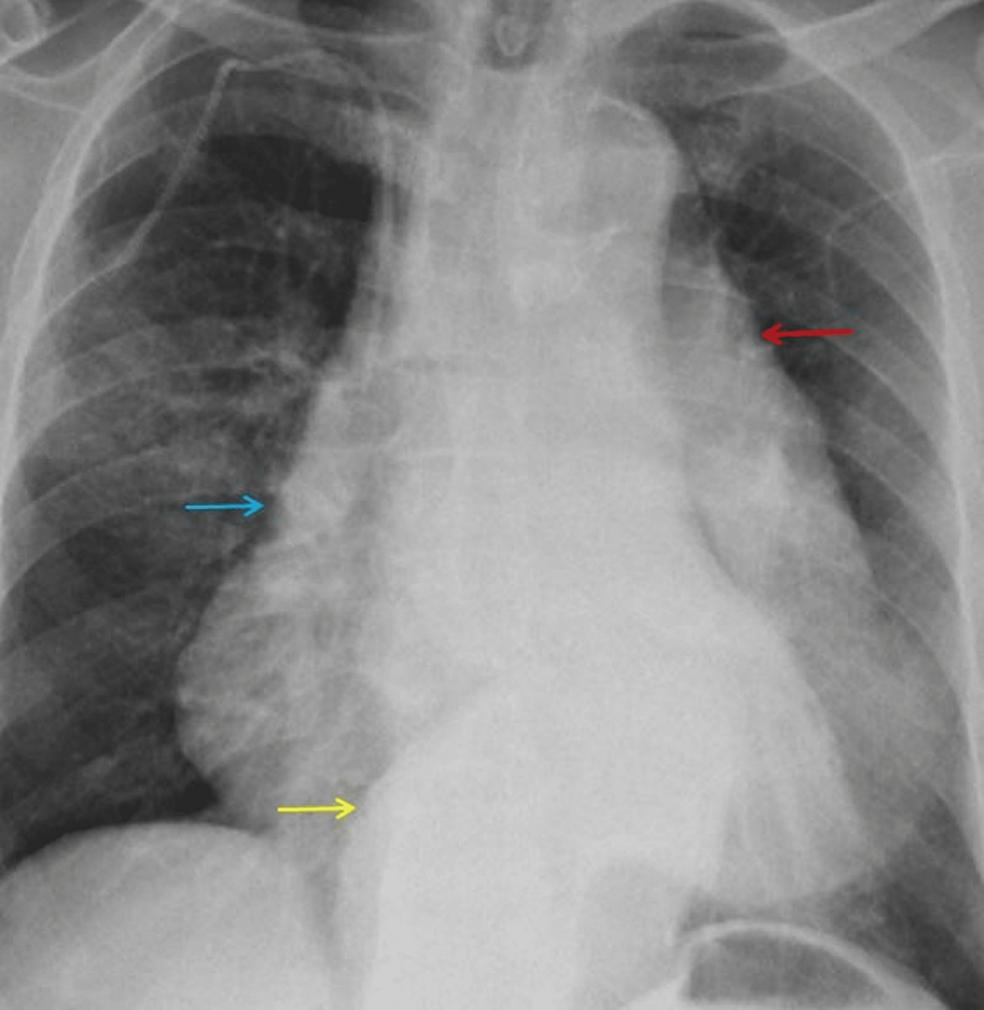
X-ray of the chest X-ray chest showing widened mediastinum (blue arrow), aortic aneurysm (yellow arrow), and obliteration of the aortic knuckle (red arrow).

Routine blood investigations like renal function and liver function tests were normal, with hemoglobin of 9 g/dL. Electrocardiogram (ECG) was normal. A chest radiograph was done, which showed widened mediastinum, obliteration of the aortic knuckle, and a large aortic aneurysm (Figure 1).

**Figure 2:**
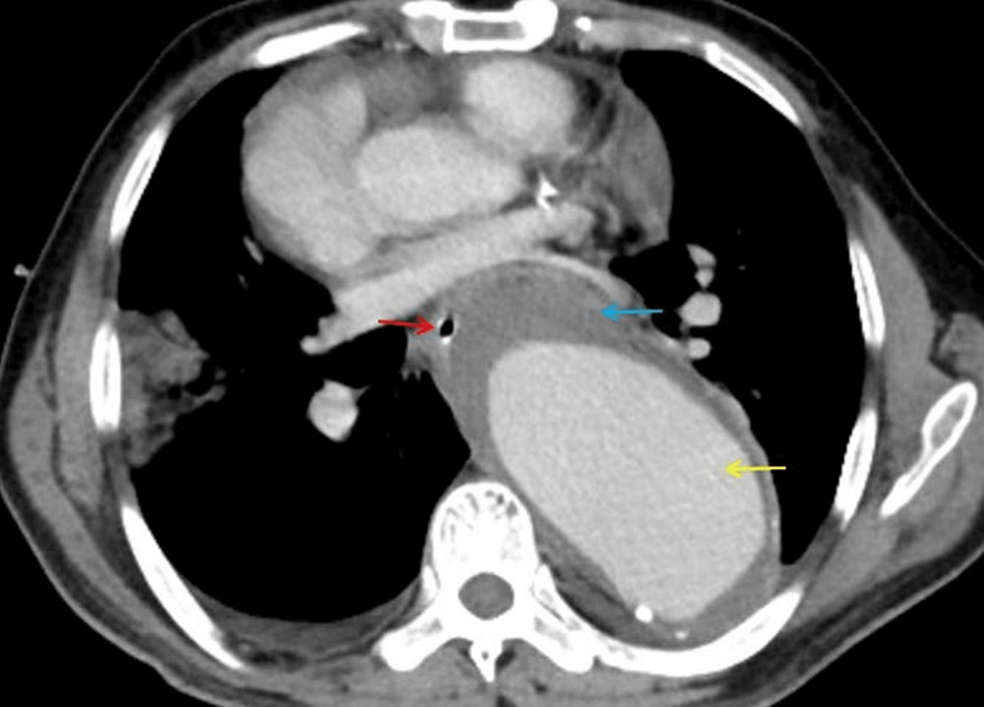
Computed tomography (axial view) of the chest Computed tomography (axial view) of the chest showing descending aortic aneurysm (yellow arrow), thrombus within the aneurysm (blue arrow) with the nasogastric tube within the esophagus (red arrow).

After stabilization, EGD was done, which showed a curvy course of the esophagus due to extraluminal compression. A minor erosion was noted in the posterolateral wall with few clots in the stomach; however, no active evidence of bleed from the varices or ulcers. Computed tomography angiography (CTA) was done, which showed a fusiform aneurysm of the arch of the aorta measuring 9 cm x 7 cm in caliber, distal to left subclavian artery origin, and descending thoracic and abdominal aorta noted up to the origin of the superior mesenteric artery. Discontinuity in the aorta wall with the possible presence of aortoesophageal fistula, which is covered by a thrombus, findings compatible with primary AEF (Figure 2).

**Figure 3:**
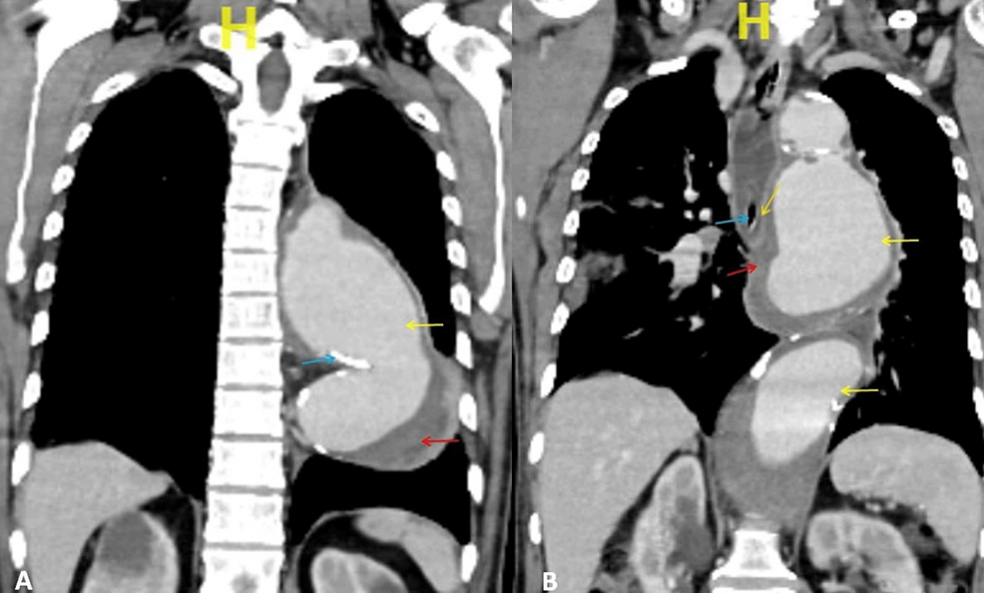
Computed tomography (coronal view) A: Descending aortic aneurysm (yellow arrow), thrombus within the aneurysm (red arrow), and atherosclerotic calcification within the wall of the aneurysm (blue arrow), and B: Descending aortic aneurysm (yellow arrow), thrombus within the aneurysm (red arrow), nasogastric tube within the esophagus (blue arrow), and the probable site of aortoesophageal fistula (orange arrow).

However, no active extravasation of the contrast material, possibly due to covering the thrombus near the fistula site. Multiple intermittent atherosclerotic plaques were noted in the thoracic and abdominal aorta. The esophagus was noted crossing the thoracic aorta anteriorly from right to left. An aortic aneurysm was noted, displacing the heart anteriorly and the esophagus right laterally (Figure 3).

The patient was planned for emergency intervention and stenting but developed massive hematemesis. The patient became unstable and hypotensive with severe tachycardia. Unfortunately, the patient expired within a few hours of the second episode of hematemesis.

## Discussion

Aortoenteric fistula is a rare fatal entity. Early clinical and imaging diagnoses with prompt surgical intervention are crucial for patient survival. There are two types of AEF, primary and secondary. A primary AEF is a sporadic disease defined as a pathologic direct connection between the aorta and any portion of the adjacent bowel in a patient without any previous aortic surgery or trauma. An AEF most commonly originates from an abdominal aortic aneurysm (AAA) due to atherosclerotic disease. Primary AEF was first described by British surgeon Sir Astley Cooper, who termed it as a ‘sometimes but serious complication of an aneurysmatic aorta’ [1]. Similarly, an aortoesophageal fistula occurs when a large thoracic aortic aneurysm compresses the esophagus in the chest. The most common cause of primary AEF is atherosclerotic aortic aneurysm, as in our case [4]. Other predisposing conditions that may lead to primary AEF include septic aortitis, mycotic infection, tuberculosis, syphilis, tumor, radiotherapy, foreign bodies, and collagen vascular diseases. Few are incidentally diagnosed through imaging, surgery, or autopsy [5].

Secondary AEF is a fatal complication after vascular surgery for an aortic disease and is more common than primary AEF. It communicates between the aorta and the adjacent bowel as a sequela of previous aortic surgery with or without a graft [5]. In both primary and secondary AEF, the third part of the duodenum is the most frequently involved site, as it is closest to the aorta [6]. In our case, the patient had aortoesophgeal fistula as the patient had a predominant thoracic aortic aneurysm.

AEF is a complex and devastating clinical condition. The AEF patients may initially present with minor herald hemorrhages, followed by a cataclysmic life-threatening gastrointestinal (GI) bleed in a later period, as in our case. The classic Chiari’s triad of symptoms includes herald hemorrhages, fatal hematemesis, and mid-thoracic pain seen in less than 45 percent of primary AEF cases [7]. Herald hemorrhages are transient, minor, and self-limiting bleeding episodes seen before the occurrence of massive life-threatening gastrointestinal bleeding hours to days later. These herald hemorrhages do not result from an actual aortoenteric communication but mucosal ulceration and focal necrosis. These appear in approximately 20 percent to 75 percent of cases [8]. The time interval between the herald and the massive bleed ranges from hours to months. Common clinical signs and symptoms of AEF patients include gastrointestinal bleeding in the form of hematemesis or haematochezia, abdominal pain, chest pain, pulsatile abdominal mass, back pain, groin mass, and sepsis. Our patient had a minor bleed followed by a massive upper gastrointestinal bleed, which led to the patient's death.

The diagnostic investigation of choice is based on the clinical condition. In a hemodynamically stable patient with gastrointestinal bleeding, EGD is the preferred primary diagnostic tool that provides valuable information [7]. However, it rarely provides confirmatory evidence of a primary AEF because stable patients do not often have active bleeding, as in our case [7]. As AEF is most commonly located in the third and fourth part of the duodenum, special attention should be paid to these areas. It may reveal ulcerations, petechiae, active bleeding, blood clots, extrinsic pulsating mass, or a portion of the graft protruding into the bowel. However, the sensitivity and specificity of endoscopy are low, as most AEF are easily missed in endoscopy because of the unclear field of vision due to accumulated blood, the experience of the endoscopist, as some endoscopists misdiagnose AEF as ulcers, erosions, polyps, and hemodynamically unstable patients cannot tolerate the procedure. In our case, EGD showed a curvy course of the esophagus due to extraluminal compression. A minor erosion was noted in the posterolateral wall with few clots in the stomach but no active evidence of bleed from the varices or ulcer.

A plain chest X-ray may show widened mediastinum, obliterated aortic knuckle, and shadow of the dilated descending aorta or aortic aneurysm, as in our case. In patients for whom intravenous contrast material is contraindicated and in unstable patients, ultrasonography (US) may be helpful. However, it is rarely indicated for diagnosing AEF, as recognizing the aberration of the deep structures on US images can be challenging [6,7].

CTA has been recommended as the favored diagnostic tool for evaluating AEF and is the first-line imaging modality for evaluating suspected AEF [2]. The predominant computed tomography findings when there is an AEF include vascular contrast within the gastrointestinal tract, ectopic gas adjacent to or within the aorta, bowel wall thickening overlying aneurysm, associated hematoma within the bowel wall or lumen or mediastinum, and the obliterated fat plane along the affected segment which usually separate the aorta from the bowel, as in our case. Oral contrast may help distinguish bowel wall thickening [9]. Nuclear medicine scans like technetium-hexametazime or indium-label white blood cell scans help detect low-grade graft infections [10,11]. In our case, CTA showed a fusiform aneurysm of the arch of the aorta extending from the left subclavian to the origin of the superior mesenteric artery with an aortoesophageal fistula, which is covered by a thrombus. The findings are compatible with the primary aortoesophageal fistula. However, no active extravasation of the contrast material, possibly due to covering the thrombus near the fistula site. 

Differential diagnosis of AEF on imaging includes retroperitoneal fibrosis (RPF), infectious aortitis, peri graft infection without fistulization, and infected (mycotic) aortic aneurysms [5]. AEF carries an extremely high mortality rate if left untreated. The mainstay of treatment for an AEF is surgery. The surgical management of primary and secondary AEF is almost similar. They are grouped into extra-anatomic bypass with aortic ligation, the gold standard approach, and in situ reconstructions [12]. Endovascular stent grafts, like a bridge or definitive therapy, may be an alternative for unstable patients and poor surgical candidates [3]. However, in situ methods of maintaining arterial perfusion have recently become popular in light of the modest outcomes following aortic stump ligation and axillo-bifemoral bypass. Sepsis is a life-threatening complication of AEF and its management, so sepsis control is crucial. We planned for emergency endovascular stenting in our patient, but unfortunately, the patient expired due to massive upper gastrointestinal bleeding.

## Conclusions

AEF is a rare cause of gastrointestinal bleeding, which can be fatal if not recognized and treated promptly. Commonly, the delay in diagnosis and management is due to a lack of knowledge regarding incidence, the common clinical presentation of AEF and the patient’s medical history, and the appropriate role of diagnostic imaging. So, the treating surgeon should have a high suspicion of AEF diagnosis if a patient presents with massive hematemesis with hypovolemic shock, with or without abdominal pain and septicemia, especially in middle-aged or elderly patients. The CT thorax and abdomen is the first-line imaging modality for detecting AEF since EGD has a low yield for diagnosis. Moreover, the treating surgeons should be aware that, though the third segment of the duodenum is the common site for AEF, other sites are also possible, such as the esophagus, as in our case. So integrated prompt management is critical with emergency exploratory surgical intervention and antibiotics with better patient post-operative care.
